# Adolescents’ ratings of features of parks that encourage park visitation and physical activity

**DOI:** 10.1186/s12966-016-0391-9

**Published:** 2016-07-04

**Authors:** Jenny Veitch, Jo Salmon, Kate Parker, Shaun Bangay, Benedicte Deforche, Anna Timperio

**Affiliations:** Institute for Physical Activity and Nutrition (IPAN), Deakin University, Geelong, Australia; School of Information Technology, Deakin University, Burwood, VIC Australia; Faculty of Medicine and Health Sciences, Department of Public Health, Ghent University, Ghent, Belgium; Faculty of Physical Education and Physiotherapy, Department of Human Biometry and Biomechanics, Vrije Universiteit Brussel, Brussels, Belgium

**Keywords:** Adolescent, Parks, Park Features, Photographic, Images, Physical activity, Perceptions

## Abstract

**Background:**

The neighbourhood environment such as the availability of parks are a key, but under-researched, influence on adolescents’ physical activity. In addition to overall physical activity levels, park-based physical activity and park visitation is low in this age group. Thus, it is critical to identify park features that may encourage or discourage adolescents from visiting parks. This study used a novel methodology to identify key physical characteristics of parks that are perceived to be important for park visitation and park-based physical activity among adolescents.

**Methods:**

Four secondary schools located in low, mid and high socio-economic status areas of Victoria, Australia were recruited. Using a purpose-built computer application, students in years 8–10 were presented with 44 original photographic images of park features. Participants rated each image (range 1–10) on how likely the feature would be to encourage them to visit a park and to engage in park-based physical activity, and placed symbols (‘thumbs up’/‘thumbs down’) on aspects of the image that had a positive or negative influence on their ratings.

**Results:**

Participants (*n* = 99) had a mean age of 13.3 years (SD = 0.87) and 53 % were female. Overall, the top three rated images prompting park visitation by adolescents were: a long steep slide, a flying fox and a table tennis table. These first two features were also reported as being likely to promote physical activity in the park. Differences in ratings were observed for boys and girls. The images that received the greatest number of “thumbs-up” symbols included large swings and slides, table tennis tables, no-smoking signs, flying foxes and BMX tracks. The images that received the greatest number of “thumbs-down” symbols included signage about rules, graffiti, toilets, concrete steps, and skate bowls.

**Conclusion:**

Physically challenging play equipment is likely to encourage adolescents to visit and be active in parks. Rules, graffiti, toilets and skate bowls may discourage visitation. It is important for park designers, planners and policy makers to consider adolescents’ views of what park design features are important so that parks are created that support and encourage visitation and optimise levels of physical activity when in the park.

**Electronic supplementary material:**

The online version of this article (doi:10.1186/s12966-016-0391-9) contains supplementary material, which is available to authorized users.

## Background

Adolescence is a key life-stage for the promotion of health behaviours as 80 % of adolescents across many countries do not achieve the recommended 60 min of daily moderate- to vigorous-intensity physical activity (MVPA) [1]. In Australia, almost one in three adolescents are overweight or obese and just 20 % are meeting physical activity recommendations [2]. Longitudinal studies have shown that a steep decline in physical activity occurs during adolescence [3] and that physical activity levels track from adolescence into adulthood [4]; thus, physical activity promotion from a young age is critical for current and future health.

The neighbourhood environment (such as the availability of parks) is an under-researched influence on adolescent physical activity, particularly as autonomy increases and adolescents have more independent access to facilities in their neighbourhood [5]. In the US, a greater availability of parks and recreational facilities close to home has been shown to be positively associated with adolescents’ physical activity. For example, in a sample of more than 4000 adolescents living in urban areas, access to a safe park within walking distance of home was positively associated with self-reported regular physical activity and negatively associated with inactivity [6]. In addition, a cross-sectional study showed that adolescent girls (*n* = 1556) who lived near more parks engaged in more objectively measured non-school MVPA compared with those with fewer parks in the neighbourhood [7]. A recent longitudinal study among adolescent girls (*n* = 730) also found that participants living within a shorter distance to a park from home were more likely to be active maintainers of objectively measured physical activity over a 3-year period [8]. However, a review of studies among youth showed associations between objectively measured access to parks and physical activity to be inconclusive [9].

A recent study observed activity levels of park visitors in Melbourne, Australia, and showed that most teens (aged 13–20 years) were observed in very low intensity activity (68 % were either sitting or standing) with only 32 % observed engaging in MVPA during observation periods [10]. It is therefore important to understand how to optimise park-based physical activity for adolescents. Differences in park visitation have also been seen for boys and girls, with boys being observed in parks more often than girls [11, 12].

It is plausible that particular park features may encourage/discourage park visitation and park-based physical activity. Little is known; however, about specific features of parks that may encourage adolescents to visit parks and what park features or conditions will encourage them to be physically active whilst in the park [7]. Most studies examining park features have been conducted with adults [13] and children [14, 15], whose findings may or may not be relevant to adolescents. Park attributes that have been shown in quantitative studies to be associated with higher levels of park use among adolescents include the presence of picnic areas, water features and basketball courts [16], playing fields [11], and playgrounds [11, 16].

The majority of studies that have examined associations between park features and physical activity among youth have examined relationships with overall or after-school physical activity and not park-based physical activity specifically. In a study of 13–15 year olds, no associations were observed between any features of the closest park and adolescent boys’ objectively measured MVPA after school. However, adolescent girls performed more MVPA after school if their closest park had trees that provided shade (six additional mins/day) and signage regarding dogs (7 mins/day) compared with girls whose nearest park did not possess these features [14]. Basketball courts [7, 12], playgrounds and walking paths [7] have also been shown to be associated with higher levels of park-based physical activity among youth.

To our knowledge, no studies have examined adolescents’ perceptions of the features they consider important for encouraging them to visit and be active in parks; despite a recognised need to determine the park features adolescents prefer [11]. Previous research has shown perceptions of park availability and quality rather than objective measures of park availability to be associated with park use and physical activity among adolescents [17]. It is important to understand adolescents’ views of what park design features are important so that parks are created that support and encourage visitation and optimise their levels of physical activity when they visit parks.

The aim of this study was to examine the physical characteristics of parks that are perceived to increase park use and park-based physical activity among adolescents, and to examine if differences were observed between girls and boys.

## Methods

This study conducted in 2014, involved the use of original digital photographs to examine features of parks that adolescents perceived to influence their park visitation and park-based physical activity. Photographic studies are a novel way to examine adolescents’ perceptions of park features [18, 19], as they do not require adolescents to visit actual park settings to view park features. In addition, by using images both the researcher and participant know exactly which feature is under consideration.

Purposive sampling was used to ensure that adolescents from a range of socio-economic status (SES) backgrounds were represented. All suburbs in Victoria were categorised into low-, middle- or high-disadvantage tertiles, using the Australian Bureau of Statistics’ Socio Economic Index for Areas (SEIFA). The SEIFA is an area-based measure of socio-economic disadvantage, constructed from the population census [20]. The ten closest suburbs within 40 km of Deakin University were chosen from each of the SEIFA tertiles and each week, three secondary schools from each tertile were selected and sent an invitation to participate in the study. After three unsuccessful attempts to make contact with the school Principal no further attempts were made and the next closest school to Deakin University within the tertile was contacted.

Overall, 35 schools were contacted and invited to participate before gaining consent from four schools (11 % response rate). The school response rate varied between SES areas. For schools located in low SES areas the response rate was 33 %, for schools located in mid SES areas the response rate was 17 % and for schools located in high SES areas the response rate was 8 %. The main reasons for non-participation included no response from the school Principal after three attempts, lack of time and schools being inundated with requests to participate in research studies. The four schools included one school from low SES, one from mid SES and two from high SES areas. Once a school was recruited, the Principal or a delegated staff member, selected two classes of year 8–10 students to complete the study protocol during a school lesson. Student packs with study information, an invitation to participate and parental consent forms were sent home to parents via the students in the selected classes (50 per school). Completed consent forms were returned for 104 of 200 students (52 %); however, five students were absent on the day of collection reducing the final sample to 99. For schools located in low SES areas the consent form response rate was 52 %, 68 % for mid SES and 38 % for schools located in high SES areas. Ethics was approved by the Deakin University Human Ethics Advisory Group (167_2013) and approval to conduct research in schools was granted from the Department of Education and Training, Victoria.

### Protocol and measures

A purpose built and designed computer application was created to enable adolescents to identify key physical characteristics of parks from original photographs of park features that may facilitate or be a barrier to park visitation and park-based physical activity. These features were identified from the literature and previous research [7, 14–16, 21, 22] and photographed from a variety of parks in different regions of Melbourne using colour digital images. The images were standardised on certain factors; for example, they were taken from eye level in dry and lightly clouded weather, and no persons were depicted. A total of 44 images were included in the final application, with each image depicting at least one specific park feature (e.g. walking path). The images were presented individually in a random order. Each image and a description of the features included in each image are presented in Additional file 1.

**Fig. 1:**
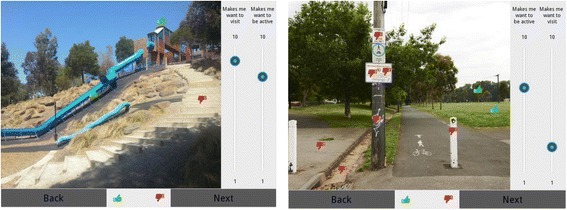
Screen shot of two park features with ‘thumbs’ symbols shown

The computer application interface included ‘sliders’ to enable students to rate each image individually using a 10-point Likert sliding scale according to how likely it was that the image ‘makes me want to visit the park’ and how likely it was that the image ‘makes me want be to be active in the park’ (1 = not likely, 10 = highly likely). The application also included symbols that could be dragged onto the image to highlight specific features within each image. Participants indicated feature(s) in each image that had the greatest influence on their rating by placing a green ‘thumbs-up’ symbol on the feature(s) that had a positive influence on their rating (see Fig. 1 for a screen-shot with ‘thumbs’ symbols shown). A similar process using a red ‘thumbs-down’ symbol was used to indicate the feature(s) that had a negative influence on their rating. Participants were able to zoom in to examine the features in more detail while also ‘panning’ (swiping side-to-side) to obtain a complete view of the image. A default setting ensured that each participant completed the rating tasks for each image before proceeding to the next image.

Once all images were rated, participants were asked to complete additional survey items to gain a general understanding of park visitation among participants and their demographic characteristics including: demographics (age and sex); usual frequency (daily, 2–3 times/week, once/week; 2–3 times/month, once/month, < once/month, not visited in past 3 months) and duration (<30 mins, 30–59 mins, 1 < 2 h, 2 < 3 h, 3 < 4 h, 4+ hrs) of park visitation in the past 3 months; usual accompaniment to parks (alone, adult family members, siblings, friends, organised group, dog, other); time taken to walk from home to the nearest park and to the park they visit most often (1–5 mins, 6–10 mins, 11–20 mins, 21–30 mins, 31+ mins, don’t know); dog ownership (yes, no); usual activities engaged in when visiting parks in the past 3 months (open ended); and three park features most likely to encourage and discourage park visitation (open ended).

The application was pilot tested with 10 adolescents prior to the study commencing and the instructions, images and survey questions were found to be suitable and easy to complete and understand. The final application was downloaded to individual Tablets. The task took approximately 15–20 min to complete and was conducted during one school class-lesson. Students were instructed on how to use the application by a research assistant.

### Data analysis

Data from each iPad were downloaded and image ratings and survey data descriptive statistics were analysed using IBM SPSS statistical software (version 22.0). Chi-square tests of independence examined differences in participant characteristics between the sexes. For each image, mean scores for ‘makes me want to visit’ and ‘makes me want to be active’ were calculated and then rankings from 1 to 44 (1 representing highest mean score) were assigned to each image for the whole sample and separately for boys and girls and frequency of park visitation. Park visitation was dichotomised as regular (≥2–3 times per month) or irregular (≤ once per month). T-tests examined significant differences between the sexes and according to frequency of park visitation. For each participant, all 44 images were then reviewed individually to count the number of ‘thumbs-up’ and ‘thumbs-down’ placed on each feature (e.g. basketball ring, slide etc.). It was not possible to limit the number of ‘thumbs’ that could be placed on particular sections of each image; however, some participants did place multiple thumbs on features. As participants were not instructed to place multiple ‘thumbs’ on a feature, this practice may not have been systematic across participants. Therefore, multiple ‘thumbs-up’ or ‘thumbs-down’ placed on a feature were only counted as one ‘thumb-up’ or ‘thumb-down’ per feature per participant.

The counts of ‘thumbs’ per feature type (e.g. swing) were summed and then divided by the number of images which included this particular feature to identify the most liked and disliked park features for the whole sample, and for boys and girls. The qualitative data from the survey items (i.e. usual activities engaged in when visiting parks, and park features most likely to encourage and discourage park visitation) were reviewed and summarised to identify the most frequently reported responses.

## Results

Table 1 presents the sample’s characteristics. The participants (*n* = 99) had a mean age of 13.3 years (SD = 0.87) and 53 % were female. Almost 40 % attended a school located in a high SES area, 34 % in a mid SES area and 26 % in a low SES area. Thirty-nine percent of participants were regular park visitors (had visited a park at least once per week over the past 3 months). Over half (53 %) reported that they usually visited parks for 1 h or less, 43 % usually visited parks with friends, 48 % could walk to their closest park in 5 min or less, 29 % could walk to the park they usually visit in 5 min or less, and 52 % owned a dog.

**Table 1:** Characteristics of participants

	Overall	Male	Female	P*
Age (*n* = 99, mean [SD])	13.3 [±0.87]	14.28 (±0.86)	14.33 (±0.91)	ns
Sex (*n* = 97, %)	-	47.4	52.6	
School attended (*n* = 97, %)				
Low SES	26.3	23.9	29.4	0.018
Mid SES	34.3	23.9	45.1	
High SES	39.4	52.2	25.5	
Usual frequency of park visit in the past 3 months (*n* = 97, %)				
≥ Once per week	38.5	45.6	29.4	ns
2–3 times per month	16.2	13.0	19.6	
≤ Once per month	33.4	28.2	39.2	
Have not visited a park in the past 3 months	12.1	13.0	11.8	
Usual duration of park visit in the past 3 months (*n* = 81, %)				
< 30 min	16.0	16.2	16.7	ns
30–59 min	37.0	32.4	40.5	
1 to <2 h	34.6	45.9	23.8	
2 or more hours	12.3	5.4	19.1	
Usual accompaniment (*n* = 97, %)^a^				
Alone	12.1	10.9	13.7	ns
Adult family members	30.3	28.3	31.4	
Brothers or sisters	27.3	21.7	33.3	
Friends	43.4	43.5	45.1	
Organised group	12.1	17.4	5.9	
Dog	23.2	19.6	23.5	
Other	5.1	2.2	7.8	
Time taken to walk from home to nearest park (*n* = 84, %)				
1–5 mins	47.7	46.2	48.9	ns
6–10 mins	25.6	23.1	26.7	
11–20 mins	17.4	23.1	13.3	
21+ mins	4.7	2.6	6.6	
Don’t know	4.7	5.1	4.4	
Time taken to walk from home to park visited most often (*n* = 81, %)				
1–5 mins	28.9	26.3	30.2	ns
6–10 mins	20.5	21.1	20.9	
11–20 mins	20.5	21.1	20.9	
21+ mins	16.8	18.5	14.0	
Don’t know	13.3	13.2	14.0	
Dog ownership (*n* = 82, %)	52.4	44.7	56.8	ns

^a^Multiple responses allowed *Chi-square tests of independence used to compare characteristics between males and females; ns denotes not significant

### Ranking of images (mean score) for ‘makes me want *to visit* the park’

The images that obtained the ten highest mean scores (overall and for boys and girls) from respondents regarding whether the image was likely to ‘make me want to visit the park’ are listed in Table 2. Overall, the highest ranked image among both boys and girls was that of a long steep slide (see Fig. 1). The second highest ranked image among both boys and girls was a flying fox, and the third ranked image overall was table tennis tables (boys ranked 3rd, girls 6th). Additional differences were observed for boys and girls. For example, images of the cement BMX track, the lake and the spider web climbing frame were ranked in the top ten for boys but not for girls, and the image of a traditional wooden swing set was ranked in the top ten for girls but not for boys. No significant differences in mean scores for visiting the park were observed between regular and irregular park visitors (data not shown).

**Table 2:** Top ten ranked images for ‘makes me want *to visit*’ the park

Images	OVERALL	BOYS	GIRLS
	Ranking	Ranking	Ranking
	(mean score[SD])	(mean score [SD])	(mean score [SD])
Gigantic blue slide (image 11)	1 (8.84 [1.93])	1 (8.74 [1.93])	1 (9.24 [1.16])
Flying fox (image 18)	2 (7.70 [2.44])	2 (7.46 [2.03])	2 (8.16 [2.48])
Table tennis tables (image 6)	3 (7.44 [2.25])	3 (7.24 [2.13])	6 (7.78 [1.19])
Big blue slide (image 10)	4 (7.32 [2.35])*	4 (6.89 [2.28])	4 (7.94 [2.05])
Big 360 swing (image 21)	5 (7.25 [2.66]) **	9 (6.43 [2.64])	3 (8.14 [2.32])
Nature-like wooden playground (image 35)	6 (7.12 [2.61]) **	8 (6.48 [2.32])	5 (7.92 [2.18])
Wooden ship with rock climbing wall, chain ladder (image 12)	7 (6.99 [2.52])	6 (6.72 [2.23])	10 (7.45 [2.54])
Concrete pathway through an attractive tree-scape (image 30)	8 (6.74 [2.69]) **	-	7 (7.65 [2.46])
Very ‘green’ looking park area with grass, trees, park bench, basketball ring (image 38)	9 (6.65 [2.60]) **	-	9 (7.55 [2.40])
Wooden playground with chrome slide, flying fox and monkey bars (image 36)	10 (6.58 [2.42])**	-	-
Cement BMX track (image 9)	-	5 (6.78 [2.39])	-
Lake in a reserve (image 3)	-	7 (6.52 [2.69])	-
Two large spider web climbing frames (image 20)	-	10 (6.39 [2.60])	-
Wooden swing set (2 adult sized swings) (image 1)			8 (7.59 [2.14])

Mean score: 1 = not likely, 10 = highly likely

Significant difference in mean score between boys and girls *p < 0.05, ** *p* < 0.01

Image numbers refer to images described in Additional file 1

### Ranking of images (mean score) for ‘makes me want *to be active* in the park’

The images that obtained the ten highest mean scores from respondents regarding whether the image was likely to ‘make me want to be active in the park’ are listed in Table 3. Overall, the highest ranked image was that of a long steep slide (see Fig. 1). This image was ranked highest for both boys and girls. The image ranked second highest overall was a flying fox (boys ranked 2nd, girls 3rd) and the image ranked third overall was another steep slide (boys ranked 8th, girls 2nd). Differences were again observed for boys and girls. For example, images of the cement BMX track, the spider web climbing frame, and the walking/cycling path were ranked in the top ten for boys but not for girls, and the image of a traditional wooden swing set was ranked in the top ten for girls but not for boys. The mean score for the image of the basketball ring (image 24) for making them want to be active in the park, was significantly higher among regular visitors compared with irregular visitors. This was the only significant difference observed according to frequency of visitation (data not shown).

**Table 3:** Top ten ranked images for ‘makes me want *to be active*’ in the park

Images	OVERALL	BOYS	GIRLS
	Ranking	Ranking	Ranking
	(mean score[SD])^a^	(mean score[SD])	(mean score[SD])
Gigantic blue slide (image 11)	1 (8.15 [1.39])	1 (7.87 [2.33])	1 (8.69 [1.97])
Flying fox (image 18)	2 (7.08 [2.69])	2 (6.80 [2.35])	3 (7.57 [2.68])
Big blue slide (image 10)	3 (6.79 [2.55])**	8 (6.02 [2.32])	2 (7.69 [2.32])
Big 360 swing (image 21)	4 (6.74 [2.86])*	5 (6.17 [2.76])	4 (7.35 [2.81])
Table tennis tables (image 6)	5 (6.71 [2.66])	3 (6.39 [2.70])	6 (7.10 [2.53])
Wooden ship with rock climbing wall, chain ladder (image 12)	6 (6.54 [2.83])**	10 (5.87 [2.57])	4 (7.35 [2.73])
Basketball ring on a line-marked court (image 24)	7 (6.35 [2.99])	6 (6.15 [3.00])	-
Nature-like wooden playground (image 35)	8 (6.33 [2.61])*	-	7 (6.94 [2.68])
Outdoor gym equipment (image 14)	9 (6.23 [2.94])	-	10 (6.78 [2.82])
Brightly painted sports goals on brick wall and ground markings (image 4)	10 (6.20 [3.14])*	-	8 (6.92 [3.04])
Cement BMX track (image 9)		4 (6.24 [2.54])	
Two large spider web climbing frames (image 20)		7 (6.09 [2.70])	
Walking/cycling path beside an oval (image 7)		9 (5.93 [2.84])	
Wooden swing set (2 adult sized swings) (image 1)			8 (6.92 [2.44])

(1 = not likely, 10 = highly likely)

Significant difference in mean score between boys and girls *p < 0.05, ** *p* < 0.01

Image numbers refer to images described in Additional file 1

### Ranking of features according to the number of ‘thumbs-up’ and ‘thumbs-down’

The features that received the most ‘thumbs-up’ in both genders included: 360 swing; table tennis tables; large slides; no-smoking signs; climbing equipment; swings (all types); flying fox; and BMX tracks (Table 4). The images that received the most ‘thumbs-down’ in both genders included: skate bowl with graffiti; concrete steps; signage with rules (e.g. no dogs allowed, dogs on leash, no loud music or no smoking); toilets; graffiti; and skate bowls without graffiti.

**Table 4:** Features with most “thumbs up” and “thumbs down” according to the number of images with feature present

	Overall	Boys	Girls
	Ranking (number of thumbs)	Ranking (number of thumbs)	Ranking (number of thumbs)
Features with most “thumbs up”			
360 swing	1 (48.0)	3 (15.0)	1 (33.0)
Table tennis tables	2 (40.0)	1 (19.0)	3 (21.0)
Large slides	3 (35.7)	2 (17.0)	5 (18.7)
No smoking signs	4 (32.5)	4 (14.0)	6 (18.5)
Climbing equipment	5 (32.0)	5 (12.7)	4 (19.3)
Swings (all types)	6 (29.4)	8 (7.7)	2 (21.7)
Flying fox	7 (27.5)	7 (11.5)	7 (16.0)
BMX tracks (all types)	8 (26.0)	6 (12.0)	8 (14.0)
Features with most “thumbs down”			
Graffitied skate bowl	1 (18.0)	2 (9.0)	1 (9.0)
Concrete steps	1 (18.0)	1 (12.0)	3 (6.0)
Signs with rules (all types)	3 (13.4)	6 (5.5)	2 (7.9)
Toilets	4 (13.0)	5 (7.0)	3 (6.0)
Graffiti (in general)	5 (12.2)	4 (7.8)	6 (4.4)
Plain concrete skate bowl	6 (11.5)	3 (8.5)	3 (6.0)

## Qualitative responses

### Usual activities engaged in when visiting the park

Adolescents reported that the most popular activities engaged in when visiting the park (in order from most to least popular) were: going for a walk or walking the dog, playing on equipment and playing games, playing sport, going for a run, talking/socialising, and riding a bike/skateboard or scooter.

### Park features most likely to encourage park visitation

The features that were reported to encourage park visitation included: swings (listed by 39 % of participants), ovals/green spaces (27 %), giant slides (19 %), basketball courts (19 %), trees (19 %), climbing equipment (15 %), and running/walking tracks (15 %). Girls were more likely than boys to report swings and trees, and boys were more likely than girls to report that ovals, climbing equipment and walking/running tracks would encourage their visitation.

### Park features most likely to discourage park visitation

The features that were reported to discourage park visitation included: signs stating that dogs were not allowed (listed by 31 % of participants), absence of playground equipment (21 %), pollution/rubbish/dirty (21 %), graffiti (17 %), skate parks (14 %), and people smoking or smoking allowed in parks (13 %). Girls were more likely than boys to report ‘no dogs allowed’ signs and pollution/rubbish/dirty and boys were more likely than girls to report that graffiti would discourage their visitation.

## Discussion

This study used novel methodology that incorporated the use of original photographic images to examine adolescents’ perceptions of the importance of park features for park visitation and park-based physical activity. Despite parks being an important setting for physical activity there is a dearth of research on specific park features associated with park use and park-based physical activity among adolescents and to our knowledge this is the first study to use photographic imagery to explore this topic among adolescents.

Overall, the features that were most likely to encourage visitation and park-based physical activity included physically challenging equipment such as: giant slides and swings, flying foxes, climbing equipment and adventure playgrounds. Facilities that encouraged fitness and ball sports such as; table tennis tables, basketball rings, outdoor gym equipment, painted markings on walls and the ground, and a concrete path with trees, were also popular. Basketball courts have previously been shown to be a popular park feature among adolescents in an observational study [16] and associated with higher levels of objectively measured non-school physical activity among adolescent girls [7].

A recent study of 12–15 year olds (*n* = 1304) living in a large rural town in Australia found that park use was associated with seven objectively measured park features: presence of a skate park, walking paths, barbeques, picnic tables, public access toilets, lighting around courts and equipment, and having more than 25 trees [23]. These features were combined to create an overall attractiveness score, where for every additional feature present, parks were almost three times more likely to be used by adolescents. None of these features were rated highly in the current study; however, features that did receive a high rating in the current study were not measured in the study by Edwards et al. The variation in results may also be due to a different assessment method, study design and being in urban versus rural location.

According to the qualitative responses, signs stating ‘no dogs allowed’ was the most common response when asked what would discourage park visitation. Signage that included statements deterring dogs such as ‘dogs not permitted in playground’ and ‘dogs not permitted on sporting ground’ also received a large number of ‘thumbs-down’. For this sample of adolescents, of whom 52 % owned a dog, being unable to visit the park with a dog appeared to be a major deterrent to park visitation. Consistent with this finding, a natural experiment in a local park that included the installation of a dog off-leash area resulted in an overall increase in park use across genders and all age groups, and an increase in the counts of park users walking and being vigorously active [24]; and dog related activities have previously been shown to be positively associated with park-based physical activity [25]. These findings highlight the potential importance of ensuring that parks do allow dogs and even provide special areas for dogs to exercise off-lead.

Although an observational study in the US found that skateboarding facilities were well attended by children 10–13 years old (especially boys) [21], the current study found that the presence of skate bowls, particularly skate bowls with graffiti, were highly likely to discourage visitation. Skateboard areas have also been shown to be negatively associated with objectively measured non-school MVPA among adolescent girls [7]. In the current study, some images of skate bowls had graffiti and some did not. It is possible that the skate parks with graffiti influenced the perceptions of the skate bowls without graffiti on occasions when the skate bowls with graffiti were shown first. This highlights the challenge of determining exactly what feature is being rated by the adolescents, and making sure that the photographs contain unambiguous images.

Fewer than half the sample were regular park visitors with almost 40 % having visited a park at least once per week in the past 3 months, with a higher percentage of boys (46 %) than girls (29 %) visiting at least once per week. Sex differences in park visitation is also likely to be a reflection of or related to sex differences in preferences of features. In addition, 43 % reported that they usually visited with friends, which reinforces that the social aspect of park visitation is important for this age group [15, 17]. It is not surprising that differences in important park features were observed for girls and boys as previous studies with youth have found activities in parks to be gender specific [16, 21]. More than half (52 %) of the boys were from schools located in high SES areas which may have had some impact on the results as previous studies in Melbourne have identified that parks in socio-economically disadvantaged areas have fewer amenities likely to promote physical activity than parks in other areas [26]. Therefore, it is possible that the boys attending the school located in the low SES area may have been more likely to be exposed to parks that had fewer or poorer quality amenities/facilities compared with the participants from schools located in high SES areas and this may have influenced their perceptions of the images presented in this study. Girls were distributed slightly more evenly across the sample with 29 % of participants from schools in low SES areas, 45 % mid SES and 26 % from schools in high SES areas. It is important to note; however, that SES was defined at the neighbourhood level according to school location. This may not be the same neighbourhood in which participants lived, and it may also not reflect the individual or family level SES of participants.

Although the present study excluded images of people, the social element of park use is important to examine in future park-based physical activity studies. Future observational studies as well experimental research such as natural experiments are needed to examine whether adolescents’ physical activity increases in the short or long-term by improving the built features of parks [27].

### Limitations

Although previous research has confirmed the validity of responses to colour images in relation to on-site responses [28], it is important to acknowledge that certain factors and features may be perceived differently in static images compared with real life situations. Thus, future studies could benefit from collecting data from adolescents in actual park settings where participants can actively experience the environment [29] and report on those experiences directly. Our results are constrained by the fact that participants were only able to rate 44 pre-determined images and it is possible that other park features may be more/less important than the features in the included images. To help counteract this limitation, as part of the survey questions at the end of the image ratings, participants were able to list any features they believed were most likely to encourage/discourage their park visitation. Interestingly, no additional features emerged from these survey items that were not already included in the 44 images. However, participants completed the survey items immediately after viewing the images and it is possible that this may have biased their responses to the survey items. In addition, some images included more than one feature in a single image (e.g. a skate bowl with graffiti) and it may have been more informative to include images that contained only one element being studied. A further limitation of the study design is the inclusion of schools in urban areas only; future studies should consider examining the perceptions of adolescents living in rural areas. Only four of the 35 schools approached, participated in the study and consent was obtained from 52 % of the students invited to participate. It is possible the low response rate may have influenced the representativeness of the schools included in the study. In addition, participating schools will not provide school-level demographic characteristics so comparisons of demographics between study participants and schools as a whole cannot be made to assist in interpretation of how representative the study sample was of the student body. Finally, the park and neighbourhood environment is likely to vary between countries therefore these findings may not be generalizable more broadly.

### Strengths

Although photography studies have examined park features with adults in relation to psychological restoration potential [18, 19], to our knowledge this is the first study to utilise original photographs to examine adolescents’ perceptions of park features that encourage visitation and park-based physical activity. Very little is known about what park features are important for adolescents, therefore obtaining input from adolescents is necessary to ensure that park design encourages visitation and physical activity among this important age group. The inclusion of girls and boys who were both regular and irregular park users are further strengths of this study as it enabled us to obtain information from participants with varied park experiences. Finally, the novel methodology utilised in this study may be transferable to studies of the built environment among other population groups.

## Conclusions

Park visitation and park-based physical activity is not common among adolescents; yet park visits and park-based physical activity could make a substantial contribution to adolescents’ overall physical activity levels. Thus, it is critical to identify park features that may encourage or discourage adolescents from making use of parks. Interest in park features are likely to change as children get older, therefore planners and park and recreation officials should carefully consider the variables that attract adolescents to parks and provide the facilities that increase parks’ attractiveness for this important age group. The findings from this study suggest that physically challenging equipment and facilities that encourage fitness and ball sports are important; however, these findings are limited by the small sample size and cross-sectional nature of the study design. This study does; however, clearly show that the photograph rating methodology used to examine park features among adolescents has excellent feasibility. It should be used in larger, more diverse and representative samples, to make it possible to identify important features and to examine the extent to which appeal for features is universal or stronger among particular sub-groups such as between boys and girls and those living in areas of varying levels of disadvantage. Experimental studies are also needed to examine whether adolescents’ physical activity increases by improving the built features of parks.

## Abbreviations

MVPA, moderate- to vigorous-intensity physical activity; SEIFA, Socio Economic Index For Areas; SES, socio-economic status

## Additional file

Additional file 1: Summary of 44 images. (DOCX 2519 kb)
