# Supplementary material for: Adolescents’ ratings of features of parks that encourage park visitation and physical activity
Source: Int J Behav Nutr Phys Act. 2016 Jul 4;13:73. doi: 10.1186/s12966-016-0391-9 (PMC4932738; doi:10.1186/s12966-016-0391-9)
Supplement: Additional file 1: — Summary of 44 images. (DOCX 2519 kb) [file 12966_2016_391_MOESM1_ESM.docx]

**Additional File 1 – Summary of 44 images**

| **Image #** | **Summary of 44 images** | **Image** |
| --- | --- | --- |
|  | Slides |  |
| 11 | - Gigantic blue slide | 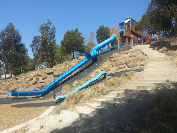 |
| 10 | - Big blue slide | 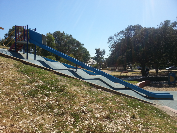 |
|  | Swings |  |
| 21 | - Big 360 swing | 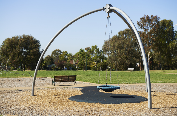 |
| 1 | - Wooden swing set (2 adult sized swings) with trees and river in background, clean and in good condition. | 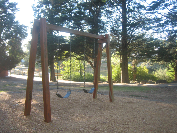 |
|  | Adventure playgrounds |  |
| 12 | - Wooden ship with rock climbing wall, chain ladder, a hole to crawl through, and a lookout tower on the top deck. | 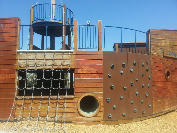 |
| 35 | - Nature-like wooden playground. Two spinning poles, bridge, walkway, chain ladder. | 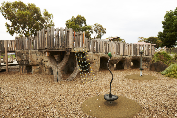 |
| 36 | - Wooden playground with some bright coloured features. Chrome slide, blue flying fox and yellow monkey bars. | 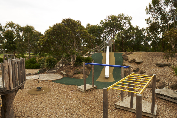 |
|  | BMX Tracks: |  |
| 9 | - Cement BMX track with bushland around. | 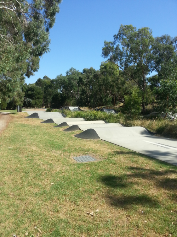 |
| 19 | - Dirt BMX track. Three visible speed bumps. | 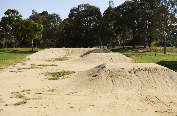 |
|  | - Skate bowls: |  |
| 17 | - Fenced off cement skate bowl with graffiti. Big playground and park area in the background. | 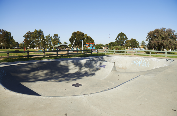 |
| 16 | - Cement skate bowl, very plain looking. Trees and grass area in the background. | 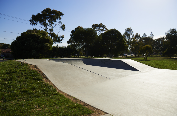 |
| 29 | - Concrete skate bowl covered in graffiti. Fence and bins in background also with graffiti. | 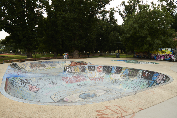 |
| 34 | - Concrete skate area with steps. Graffiti is all over the area. BBQ area behind with a large permanent shelter. | 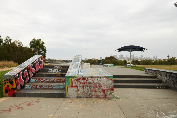 |
|  | Basketball rings: |  |
| 24 | - Basketball ring on a line-marked court. | 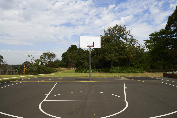 |
| 5 | - Standalone basketball ring on a blue basketball court surface, reserve in background. | 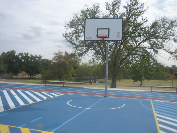 |
| 25 | - Basketball and netball ring. One park bench visible, area fenced off with half stone, half metal fence. | 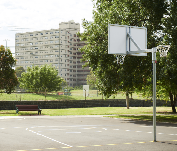 |
| 15 | - Basketball and netball ring on an old looking cement court surface. Shed on the side covered in graffiti. Two park benches, a walking path, trees, grass, and street in the background. | 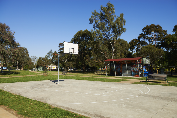 |
|  | Outdoor gym equipment: |  |
| 14 | - Outdoor gym equipment, orange and grey | 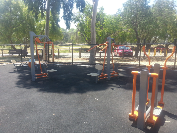 |
| 8 | - Outdoor gym equipment with instruction board. Black and chrome only. | 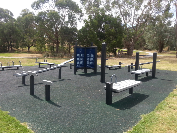 |
|  | Climbing Frames: |  |
| 20 | - Two large spider web climbing frames. | 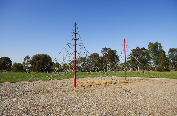 |
| 27 | - Spider web climbing equipment. Ropes on the outside are red, tanbark underneath. Bushes and trees behind and outside fence is visible. | 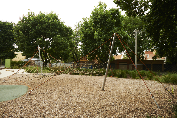 |
|  | Paths: |  |
| 7 | - Walking/cycling path on the side of a fenced off oval. 2 park benches, 4 lights and trees are visible along the path | 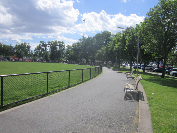 |
| 41 | - Park with a walking path, colourful and wooden playground, basketball ring, park bench and table, bins, trees and grassy area. | 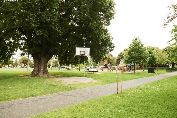 |
| 31 | - Concrete pathway through a grassy park area with lots of trees. Fenced off area far left with bins visible. Sign painted on path to indicate cycling allowed. | 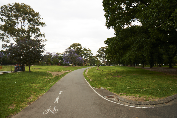 |
| 30 | - Concrete pathway through an attractive tree-scape. Very well maintained grass and tanbark areas around the walkway. | 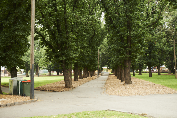 |
|  | - Ovals: |  |
| 22 | - Green grass oval. One light pole, football goals, cricket nets and trees visible behind the oval. | 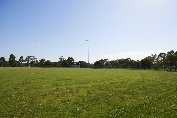 |
| 32 | - Plain looking soccer oval with goal posts. | 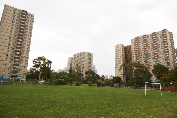 |
| 28 | - Sports oval. Water fountain, graffiti on rubbish bins and a walking path are visible at the front. | 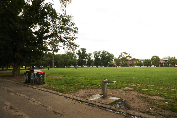 |
| 42 | - Sports oval, very short cut grass, stadium/stands in background, oval is fenced off with a wire fence, sign on the fence: No dogs on sporting ground sign. | 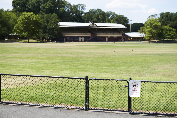 |
|  | Signage: |  |
| 39 | - Sign post in the middle of the image: dogs must be leashed in these gardens. | 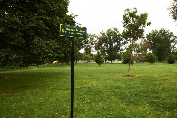 |
| 26 | - Post with three signs: think helmet, shared footway, no loud music in parks. Walking/cycling path goes through a grassy park area. Graffiti on posts. | 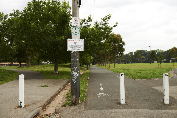 |
| 44 | - Signs on the fence stating: no dogs and no smoking. Graffiti on a post and bins visible behind the fence. | 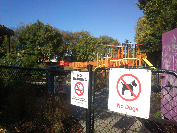 |
| 40 | - Sign saying: that it is a smoke-free playground. Walking path in the middle with a grassy area including a table and bench and a couple of trees. A well maintained, clean park | 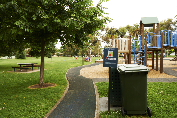 |
| 33 | - Sign saying: dogs not permitted in playgrounds at any time, penalties apply. | 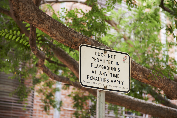 |
| 38 | Very 'green' looking park area:   - Very green looking park area with grass, trees, park bench, basketball ring and a cottage in the background, all looks very well maintained. | 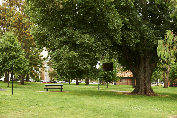 |
| 3 | Lake:   - Lake in a reserve, one park bench to the right and bridge at the back. All clean and well maintained. | 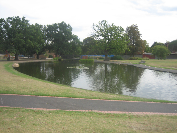 |
| 6 | Table tennis tables:   - Brightly coloured | 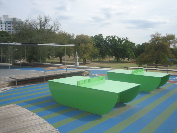 |
| 18 | Flying fox   - Flying fox | 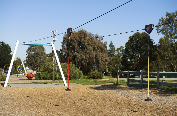 |
| 13 | Play equipment:   - Blue and grey balance feature. Small pad like circles connected by rope for youth to try and balance as they cross. Playground visible in the background with naughts and crosses, colourful stepping stones, chain ladders and bridges. | 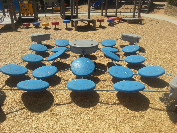 |
| 4 | Painted markings:   - Brightly painted sports goals on brick wall and painted ground markings. | 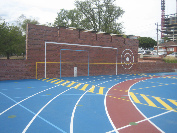 |
| 43 | Bike rack:   - Bike rack with bikes, then a fenced off colourful playground behind with the swing set visible | 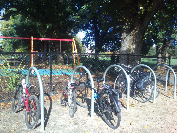 |
| 37 | BBQ:   - Wooden table and benches and a covered area with more tables and benches behind. Three visible bins which have graffiti. Grass area and walking track visible behind. | 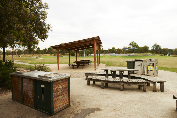 |
| 23 | Cricket nets:   - Half cement, half grass pitch in decent condition. | 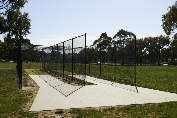 |
| 2 | Toilet:   - Toilet block with concrete bike/walking path, trees and grass visible. Toilets in good condition with no visible graffiti/litter | 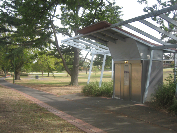 |
